# Impaired Glucose Metabolisms of Patients with Obstructive Sleep Apnea and Type 2 Diabetes

**DOI:** 10.1155/2018/6714392

**Published:** 2018-12-23

**Authors:** Ye Zhang, Yanpeng Xing, Haibo Yuan, Xiaokun Gang, Weiying Guo, Zhuo Li, Guixia Wang

**Affiliations:** ^1^Department of Endocrinology & Metabolism, The First Hospital of Jilin University, Changchun, Jilin, China; ^2^Department of Gastrointestinal Surgery, The First Hospital of Jilin University, Changchun, Jilin, China; ^3^Department of Respiratory, The First Hospital of Jilin University, Changchun, Jilin, China

## Abstract

**Aims:**

Obstructive sleep apnea (OSA) is a very common disorder which is associated with metabolic comorbidities. The aims of this study were to analyze clinical data of patients with OSA and evaluate influence of sleep-disordered breathing on glycometabolism and its underlying mechanisms.

**Methods:**

We designed a cross-sectional study involving 53 OSA patients in The First Hospital of Jilin University from March 2015 to March 2016. They underwent a full-night polysomnography, measurement of fasting blood glucose and blood lipid profiles. Besides, we chose 20 individuals with type 2 diabetes mellitus (T2DM) as a subgroup for an in-depth study. This group additionally underwent a steamed bread meal test and measurement of HbA1c, C-reactive protein, tumor necrosis factor-*α*, interleukin 6, morning plasma cortisol, and growth hormone.

**Results:**

The two groups which with or without T2DM showed no significant differences in baseline characteristics. As for OSA patients with T2DM, the severe OSA group had higher homeostasis model assessment of insulin resistance (HOMA-IR) (*P* = 0.013) than the mild-to-moderate OSA group, whereas had lower morning plasma cortisol levels (*P* = 0.005) than the mild-to-moderate OSA group. AHI was positive correlated with HOMA-IR (*r* = 0.523, *P* = 0.018), yet negative correlated with morning plasma cortisol (*r* = −0.694, *P* = 0.001). However, nadir SpO_2_ was positive correlated with morning plasma cortisol (*r*_s_ = 0.646, *P* = 0.002), while negative correlated with HOMA-IR (*r*_s_ = −0.489, *P* = 0.029).

**Conclusions:**

Our study showed that sleep-disordered breathing exerted negative influence on glucose metabolisms. The impairment of hypothalamic-pituitary-adrenal axis activity may be one of the underlying mechanisms of the glycometabolic dysfunctions in OSA with T2DM patients.

## 1. Introduction

Obstructive sleep apnea (OSA), a common sleep-related breathing disorder, is characterized by repetitive upper airway narrowing during sleep and recurrent arousal from sleep that leads to sleep fragmentations. OSA affecting 10–25% of the general population worldwide is more likely to combine with metabolic dysfunctions [1, 2].

Increasing evidence has showed that OSA is associated with type 2 diabetes mellitus (T2DM) and may exert negative influence on glucose metabolisms [2–4]. Several large cohort studies have found a high prevalence of insulin resistance and T2DM in OSA patients, independent of age and obesity [5, 6]. Furthermore, severity of OSA is associated with development of insulin resistance, glucose intolerance, and T2DM [7, 8]. However, underlying mechanisms regarding relationship between OSA and T2DM have not been thoroughly explored.

The hallmarks of OSA, namely, sleep fragmentation and intermittent hypoxia, may contribute to impaired glucose metabolism by multifactorial process including sympathetic nerve activation, oxidative stress, inflammation, and hormonal changes [1]. Moreover, other mechanisms involving gut microbiota dysbiosis and endoplasmic reticulum stress are still under investigation [2].

Several studies have showed that OSA activates alterations in hormone secretory patterns [9]. Reciprocally, the change of cortisol and growth hormone levels may lead to impaired glucose metabolisms [1, 10]. With respect to alterations in hypothalamic-pituitary-adrenal (HPA) activity, some studies found an increased nocturnal pulsatile cortisol release which may result in insulin resistance [11, 12], while others reported a decreased HPA axis activity presenting lower morning cortisol [13]. Another study also demonstrated lower cortisol responses in OSA patients than subjects without OSA [14]. Likewise, based on previous studies, it is evident that obese patients with OSA have impaired basal and stimulated growth hormone secretion [15]. In addition, growth hormone inhibits insulin activity and usually mounts in the first half of the sleep inducing impaired glucose metabolisms [16].

Except for hormonal alterations, inflammation response is the main pathogenesis of OSA-associated metabolic processes through the activation of inflammatory pathways [17, 18]. A meta-analysis has showed higher levels of C-reactive protein (CRP), tumor necrosis factor-*α* (TNF-*α*), and interleukin 6 (IL-6) in patients with OSA compared to the control group [19], albeit inconsistent results reported by previous studies. OSA patients are in the condition of inflammation response leading to impaired glucose metabolisms [1]. Therefore, increasing studies on inflammatory pathways illuminate mechanisms of inflammatory response in OSA patients [1, 20].

To date, substantial studies have expanded our knowledge of relationship between OSA and T2DM. However, it is important to highlight that animal model could not involve all the major components of OSA patients due to technical difficulties. Thus, studies based on OSA patients to some extent seem to be more convincing. In this regard, we designed a cross-sectional study enrolling patients with OSA to investigate altered glucose metabolisms in OSA. Besides, we intended to apply clinical data to explore underlying mechanisms of impaired glucose metabolisms in OSA. Our in-depth elucidation on effect of sleep-disordered breathing on glucose metabolisms might be beneficial for patients with OSA and T2DM to make preventive and therapeutic strategies.

## 2. Materials and Methods

### 2.1. Subjects

A total of 53 patients were enrolled according to the following inclusion and exclusion criteria. All patients were newly diagnosed OSA at Respiratory Sleep Center in The First Hospital of Jilin University from March 2015 to March 2016. The diagnosis of OSA was in accordance with the guidelines of the American Academy of Sleep Medicine (AASM) [21]. Inclusion criteria were (1) newly diagnosed OSA; (2) at the age of 20-70 years old; and (3) diabetic duration less than 10 years and glycemic control by diet, exercise, or medication for at least 3 months. Exclusion criteria were (1) diagnosed viral hepatitis, cancer, chronic obstructive pulmonary disease, asthma, and other pulmonary diseases and (2) history of OSA treatment prior to the study.

Patients were classified into two groups based on diagnosis of T2DM: the non-T2DM group (*N* = 33) and the T2DM group (*N* = 20). In order to further investigate the relationship between OSA and T2DM, we defined patients with OSA and T2DM as a subgroup. Then, we divided the subgroup into two groups based on apnea-hypopnea index (AHI) and severity of OSA: the mild-to-moderate OSA group (5 ≤ AHI ≤ 30 events/h, *N* = 7) and severe OSA group (AHI > 30 events/h, *N* = 13).

All the participants enrolled in this study underwent a medical history questionnaire, physical examination. Body weight, height, waist circumference, neck circumference, and arterial blood pressure were measured in the morning.

The study was approved by the research ethics committee of The First Hospital of Jilin University. Informed consent was obtained from all participants included in this study.

### 2.2. Sleep Assessment

Full-night polysomnography (PSG) was performed in the sleep center (Alice 5LE, Philips Respironics). All data were manually scored and evaluated in accordance with guidelines of AASM. The duration of sleep study lasted more than 6 hours. The definitions of apnea, hypoxia, and oxygen desaturation index (ODI) were described as follows. Apnea was defined as a complete cessation of respiratory airflow more than 10 s. Hypopnea was defined as a decrease in airflow by more than 50% from baseline for at least 10 s, in association with a reduction in oxygen saturation of at least 3%. ODI was defined as a number of arterial oxygen saturation (SpO_2_) reduction of 3% or more per hour. Total sleep time (TST), sleep efficiency, AHI, nadir SpO_2_, ODI, and other polysomnographic parameters were measured and recorded after an overnight PSG.

### 2.3. Blood Sampling and Laboratory Testing

After an overnight fasting of at least 8 h, a venous blood sample was collected to measure the fasting blood glucose and lipid profile. As for the patients with OSA and T2DM, they additionally examined HbA1c, CRP, TNF-*α*, IL-6, morning plasma cortisol, and growth hormone and underwent a steamed bread meal test. Blood glucose, insulin, and C-peptide levels were determined at 0 and 120 min during a steamed bread meal test. Insulin resistance was assessed by homeostasis model assessment of insulin resistance (HOMA-IR) calculated as fasting serum insulin (mU/mL) multiplying fasting blood glucose (mmol/L) divided by 22.5 [22]. Levels of insulin, C-peptide, CRP, TNF-*α*, IL-6, morning plasma cortisol, and growth hormone were measured by enzyme-linked immunosorbent assay (ELISA) at the same clinical laboratory.

### 2.4. Statistical Analysis

Statistical analyses were carried out using the SPSS 22.0. Continuous data with normal distribution were expressed as mean ± standard deviation (SD), continuous data with nonnormal distribution were expressed as median (first quartile, third quartile), and noncontinuous data were expressed in percentage. Statistical comparisons were performed using a *t*-test, Mann-Whitney *U* test, or chi-square test. Use Spearman or Pearson correlation to perform correlation analysis. The statistical significance was set at *P* < 0.05.

## 3. Results

The study consisted of 53 OSA patients which were divided into two groups, the non-T2DM group and T2DM group. Baseline characteristics of the subjects are presented in Table 1. There were no statistically significant differences in anthropometric parameters, comorbidities, sleep characteristics, and lipid profiles between the groups. However, fasting blood glucose level was significantly higher in the T2DM group than non-T2DM group.

To further investigate the relationship between OSA and T2DM, we defined patients with OSA and T2DM as a subgroup. Then, we divided it into two groups based on the severity of OSA, the mild-to-moderate OSA group (5 ≤ AHI ≤ 30 events/h) and severe OSA group (AH > 30 events/h).

The baseline characteristics of OSA and T2DM patients are presented in Table 2. There were no statistically significant differences in gender, age, and anthropometric parameters between the mild-to-moderate OSA and severe OSA groups. With respect to the characteristics of T2DM, the duration, therapies, and chronic complications of T2DM showed no statistically significant differences between the groups which are presented in Table 3.

The analyses of polysomnographic data of OSA with T2DM patients are presented in Table 4. AHI and ODI were significantly lower in the mild-to-moderate OSA group compared to the severe OSA group, while stage N3/TST, nadir SpO_2_, and mean SpO_2_ were higher in the mild-to-moderate OSA group. Moreover, there were no significant differences in TST, sleep efficiency, stage N2/TST, and stage rapid eye movement (REM)/TST.

The results of biochemical parameters of OSA with T2DM patients are showed in Table 5. As for glucose metabolic parameters, the severe OSA group showed higher HOMA-IR and insulin levels than the mild-to-moderate OSA group. In order to further study the mechanisms of impaired glucose metabolism of OSA and T2DM patients, we examined CRP, TNF-*α*, IL-6, morning plasma cortisol, and growth hormone levels. The results showed no significant differences between the groups except for morning plasma cortisol. The severe OSA group had significantly lower morning plasma cortisol levels compared to the mild-to-moderate OSA group. In addition, there were no significant differences in lipid profiles between the groups.

**Figure 1:**
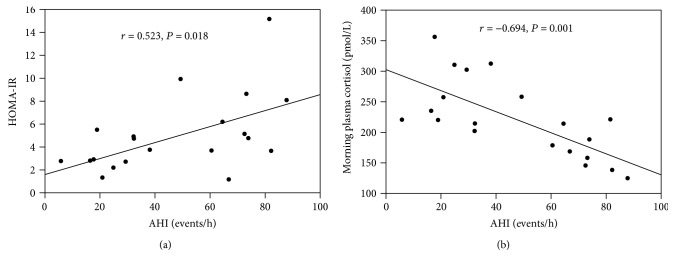
Correlation between AHI and HOMA-IR (a) and correlation between AHI and morning plasma cortisol (b) of OSA with T2DM patients. HOMA-IR homeostasis model assessment of insulin resistance. AHI: apnea-hypopnea index.

**Figure 2:**
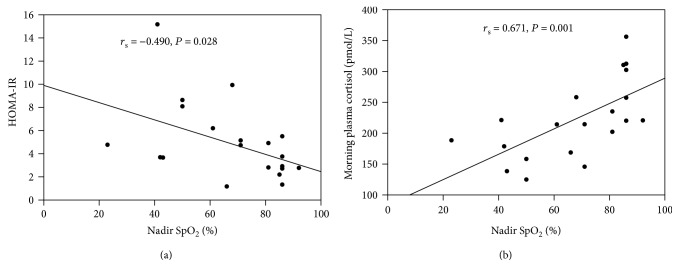
Correlation between nadir SpO2 and HOMA-IR (a) and correlation between nadir SpO2 and morning plasma cortisol (b) of OSA with T2DM patients. HOMA-IR homeostasis model assessment of insulin resistance. AHI: apnea-hypopnea index.

In the correlation analysis of OSA and T2DM patients, AHI was significantly positive correlated with HOMA-IR (*r* = 0.523, *P* = 0.018), yet negative correlated with morning plasma cortisol (*r* = −0.694, *P* = 0.001) (Figure 1). However, nadir SpO_2_ was significantly positive correlated with morning plasma cortisol (*r*_s_ = 0.646, *P* = 0.002), while negative correlated with HOMA-IR (*r*_s_ = −0.489, *P* = 0.029) (Figure 2).

## 4. Discussion

This study analyzed clinical data of OSA patients to investigate the relationship between OSA and T2DM. Moreover, we defined patients of OSA and T2DM as a subgroup to study the influence of OSA on glucose metabolisms and its underlying mechanisms. Accumulative evidence has showed that OSA is independently associated with T2DM and has negative influence on glucose metabolisms [2–4]. However, studies yielded inconsistent results and related mechanisms remained to be further investigated.

In our study, 53 patients were enrolled based on the inclusion and exclusion criteria. The mean age of OSA patients was 45.42 ± 11.26 years and male to female ratio was 3.42 : 1. The prevalence of T2DM, obesity, hypertension, dyslipidemia, and nonalcoholic fatty liver disease took up 37.74%, 64.15%, 77.36%, 79.25%, and 94.34%, respectively. There were no significant differences in metabolic comorbidities between the non-T2DM group and T2DM group. Several epidemiological studies have suggested that OSA is an independent risk factor for the development of T2DM [23]. Furthermore, insulin resistance is a key factor in the pathogenesis of T2DM and other OSA-associated metabolic perturbations [1]. As for patients with OSA and T2DM, the effects of sleep-disordered breathing on glucose metabolisms and underlying mechanisms have not been thoroughly demonstrated. Therefore, we had a further study on relationships between OSA severity and glucose metabolisms.

The results of glucose metabolism disorders in our study suggested that the severe OSA group had higher HOMA-IR and insulin levels than the mild-to-moderate OSA group independent of age, gender, obesity, diabetic duration, and antidiabetic therapies. In line with these results, epidemiologic studies have proved an association of OSA and impaired glucose metabolism after adjustments of known confounders. Several cross-sectional studies found that OSA was associated with increased insulin resistance in spite of adjustment for obesity and other confounders [24–26]. Besides, sleep-disordered breathing may also contribute to poor diabetic control. Analyses of the European Sleep Apnea Database showed that OSA severity was related to increased HbA1c levels [27, 28]. However, we did not find significant differences in HbA1c and other glycometabolism between the groups. Considering the factors that all subjects had glycemic control by diet, exercise, or drugs for at least three months before enrolled in our study, plus the relatively small sample size, it seems reasonable to understand the inconsistent results on glycemic control.

To investigate if sleep-disordered breathing has an impact on HPA axis, we further examined plasma cortisol levels and then showed that the severe OSA group had significantly lower morning plasma cortisol levels compared to the mild-to-moderate OSA group. Additionally, severity of sleep fragmentation and intermittent hypoxia correlated with HOMA-IR in OSA with T2DM patients.

The effects of sleep-disordered breathing on the HPA axis and morning cortisol levels remain controversial. Some studies have reported no significant association [29–31] or increased HPA axis activity [32, 33], whereas others reported decreased HPA axis activity compared to controls [13, 14]. The inconsistent findings are partly due to obesity, timing of sample collection, and other possible confounders. As for morning plasma cortisol levels, some studies found that there are no significant differences in morning plasma cortisol levels [29, 34], while others showed lower morning cortisol levels than control subjects [13]. There are two possible explanations of lower morning plasma cortisol levels regarding change of HPA axis activity. Considering the temporary inhibition of cortisol caused by pulsatile cortisol release associated with nocturnal awakenings [35], OSA patients had lower morning plasma cortisol levels compared to normal individuals. Moreover, sleep-disordered breathing exacerbated negative feedback effect on the HPA axis which results in lower morning plasma cortisol levels. In parallel with our findings, Bozic et al. reported negative association between severity of OSA and morning plasma cortisol levels [13]. Therefore, our study suggested that HPA axis activity gradually decreased as severity of OSA became worse.

Although changes of HPA axis in patients with OSA present different results, they mostly exhibit nocturnal hypercortisolism which is responsible for the negative effects on glucose metabolism [1, 36, 37]. Previous studies have showed that SA patients had higher nocturnal cortisol levels compared to control subjects [11, 12]. In line with the elevation of late-night serum cortisol, 24-hour urinary cortisol levels were also higher in OSA patients, indicating that night wakefulness boosts the activity of HPA axis and increases pulsatile cortisol release. Furthermore, Plat et al. reported that boosted nocturnal cortisol levels contribute to alterations in glucose tolerance, insulin sensitivity, and insulin secretion [10]. Our study showed positive correlation between AHI and HOMA-IR, whereas negative correlated with morning plasma morning cortisol. It suggested that impaired HPA axis activity may lead to insulin resistance, albeit devoid of data on nocturnal cortisol levels.

In our study, we did not find difference in morning growth hormone between the mild-to-moderate OSA group and severe OSA group. Nevertheless, based on previous elaboration, it is evident that obese patients with OSA showed an impairment of both basal and stimulated growth hormone secretions [15]. Sleep fragmentation and intermittent hypoxia negatively affect secretion of growth hormone [9]. Reciprocally, growth hormone inhibits insulin activity and usually peaks in the first half of the sleep period. Sleep restriction might be associated with elevation of growth hormone secretion at night causing impaired glucose metabolisms [16].

Admittedly, studies have showed that OSA induces changes in the levels or secretory patterns of several hormones. However, vascular and systemic inflammation is the main pathogenesis of OSA-associated cardiometabolic processes through the activation of inflammatory pathways [17, 18, 20]. Our study showed no differences in CRP, TNF-*α*, and IL-6 between the mild-to-moderate OSA group and severe OSA group. Increasing studies have addressed the increased levels of various circulating biomarkers of inflammation, and results have been diverse and heavily confounded by obesity. A meta-analysis of 51 studies showed higher levels of CRP, TNF-*α*, and IL-6 in patients with OSA compared to controls [19]. Although we found no differences in CRP, TNF-*α*, and IL-6 between the groups, it did not accurately implicate levels of inflammatory responses in other organs and tissues. Previous studies showed that intermittent hypoxia precipitates inflammatory response which has a detrimental effect on multiple systems, also leading to impaired glucose metabolisms [1]. Hypoxia-sensitive transcriptional factors, hypoxia-inducible factor-1 (HIF-1) and nuclear factor-*κ*B (NF-*κ*B), might mediate the inflammatory consequences of OSA [20, 38]. It is likely that crosstalk between NF-*κ*B and HIF-1 plays a complex role in modulating the inflammatory response to intermittent hypoxia in OSA [39–41]. Therefore, there is no denying that multiple inflammatory mediators play key roles in the mechanisms of glucose metabolic dysfunctions.

An increasing number of studies have demonstrated an independent association between OSA, insulin resistance, and T2DM [28, 42]. Furthermore, mounting evidence has showed a link between OSA severity and development of insulin resistance and T2DM [7, 8]. In our study, the severe OSA group had higher HOMA-IR and insulin levels in patients with OSA and T2DM independent of age, gender, obesity, diabetic duration, and antidiabetic therapies. We also found that as sleep-disordered breathing became worse, evaluated by AHI and nadir SpO_2_, the level of HOMA-IR increased. The impact of sleep-disordered breathing on glucose metabolisms seems to be insidious, but also harmful for patients with T2DM. In this regard, we attempt to reveal potential mechanisms of glucose metabolic dysfunctions in OSA with T2DM patients. As previously discussed, inhibition of morning plasma cortisol appears to be a manifestation of impaired HPA axis and nocturnal hypercortisolism might be responsible for insulin resistance. Therefore, we concluded that the impairment of HPA axis activity may explain the underlying mechanisms of the glycometabolic dysfunctions in OSA. Although we did not find significant differences in growth hormone, CRP, TNF-*α*, and IL-6 between the groups, inflammatory response in OSA might play a key role in impaired glucose metabolism based on previous studies. In addition, other mechanisms including sympathetic nerve activation and oxidative stress might integrally contribute to insulin resistance and T2DM.

There are a few limitations in our study. It might not sufficient to examine hormone levels at a single point. Instead, a 24-hour cortisol profile could provide more convincing results of HPA axis activity. Moreover, continuous glucose monitoring combined with PSG may be of additional value to study the effect of sleep-disordered breathing on nocturnal glycemic variations which is also beneficial to glycemic control for patients with T2DM. Besides, considering the relatively small sample size of our study, larger number of studies, especially cohort studies, would confirm the findings of this study.

In conclusion, our study showed that sleep-disordered breathing exerted negative influence on glucose metabolisms. The severe OSA group had significantly higher HOMA-IR, yet lower morning plasma cortisol levels than the mild-to-moderate OSA group. Furthermore, severity of OSA is positively correlated with HOMA-IR, whereas negatively correlated with morning cortisol levels. Although underlying mechanisms of association between OSA and impaired glucose metabolisms remain unclear, the change of HPA axis activity may be involved in the pathophysiological mechanisms among patients with OSA and T2DM. Further studies with larger sample size and more sufficient data are needed to confirm these findings.

## Figures and Tables

**Table 1:** Characteristics of OSA with non-T2DM and OSA with T2DM.

Parameters	Non-T2DM (*N* = 33)	T2DM (*N* = 20)	*P*
Male	24 (72.73)	17 (85.00)	0.486
Female	9 (27.27)	3 (15.00)	
Age (years)	44.03 ± 11.60	47.70 ± 10.57	0.254
Weight (kg)	84.27 ± 13.58	87.94 ± 11.69	0.321
Height (m)	1.71 ± 0.08	1.73 ± 0.06	0.248
BMI (kg/m^2^)	28.86 ± 3.50	29.36 ± 2.94	0.597
Waist circumference (cm)	105.00 (89.50, 108.00)	102.00 (97.00, 109.00)	0.508
Neck circumference (cm)	45.00 (39.00, 48.00)	45.00 (41.00, 46.00)	0.804
Comorbidities			
Obesity	19 (57.58)	15 (75.00)	0.200
Hypertension	28 (84.85)	13 (65.00)	0.182
Dyslipidemia	24 (72.72)	18 (90.00)	0.249
NAFLD	30 (90.91)	20 (100.00)	0.438
Sleep characteristics			
TST (min)	382.12 ± 59.48	391.60 ± 51.00	0.556
Sleep efficiency (%)	82.80 ± 12.25	83.76 ± 9.51	0.768
Stage N1/TST (%)	17.88 ± 11.92	21.34 ± 13.66	0.337
Stage N2/TST (%)	62.23 ± 10.10	61.83 ± 15.25	0.910
Stage N3/TST (%)	0 (0, 5.95)	0 (0, 2.81)	0.787
Stage REM/TST (%)	17.11 ± 6.71	15.92 ± 4.65	0.487
AHI (events/h)	31.60 (19.55, 72.05)	43.70 (21.90, 73.03)	0.633
N-REM AHI (events/h)	27.60 (18.25, 73.05)	40.25 (22.53, 74.90)	0.430
REM AHI (events/h)	53.90 (22.45, 65.25)	48.45 (25.13, 63.83)	0.577
Nadir SpO_2_ (%)	82.00 (73.00, 87.00)	74.00 (50.50, 86.00)	0.139
Mean SpO_2_ (%)	95.00 (93.00, 96.35)	93.00 (87.50, 95.88)	0.075
ODI (events/h)	21.40 (11.00, 73.80)	38.30 (17.50, 73.68)	0.271
Biochemical parameters			
FBG (mmol/L)	5.41 ± 0.58	8.82 ± 1.86	≤0.001^∗^
Cholesterol (mmol/L)	4.84 ± 0.73	4.44 ± 1.04	0.111
Triglycerides (mmol/L)	2.25 ± 1.32	2.30 ± 0.91	0.889
HDL cholesterol (mmol/L)	1.14 ± 0.22	1.07 ± 0.18	0.221
LDL cholesterol (mmol/L)	3.09 ± 0.68	2.84 ± 0.85	0.245

Continuous data with normal distribution are presented as mean ± SD, continuous data with nonnormal distribution are presented as median (first quartile, third quartile), and noncontinuous data are presented as number (%). T2DM: type 2 diabetes mellitus; BMI: body mass index; NAFLD: nonalcoholic fatty liver disease; TST: total sleep time; AHI: apnea-hypopnea index; REM: rapid eye movement; N-REM: nonrapid eye movement; SpO_2_: arterial oxygen saturation; ODI: oxygen desaturation index; FBG: fasting blood glucose; HDL: high-density lipoprotein; LDL: low-density lipoprotein (^∗^*P* < 0.05).

**Table 2:** Baseline characteristics of OSA with T2DM patients.

Parameters	Mild-to-moderate OSA (*N* = 7)	Severe OSA (*N* = 13)	*P*
Male	6 (85.71)	11 (84.62)	0.730
Female	1 (14.29)	2 (15.38)	
Age (years)	43.29 ± 6.40	51.38 ± 10.03	0.070
Weight (kg)	95.00 (83.00, 98.00)	82.00 (76.00, 95.15)	0.203
Height (m)	1.75 ± 0.04	1.72 ± 0.07	0.266
BMI (kg/m^2^)	30.13 ± 2.65	28.94 ± 3.11	0.402
Waist circumference (cm)	103.00 (100.00, 110.00)	102.00 (94.00, 110.00)	0.781
Neck circumference (cm)	43.57 ± 3.17	43.77 ± 2.80	0.887

Continuous data with normal distribution are presented as mean ± SD, continuous data with nonnormal distribution are presented as median (first quartile, third quartile), and noncontinuous data are presented as number (%). BMI: body mass index.

**Table 3:** Baseline characteristics of T2DM.

Parameters	Mild-to-moderate OSA (*N* = 7)	Severe OSA (*N* = 13)	*P*
Duration of T2DM (months)	36.00 (24.00, 60.00)	36.00 (18.00, 114.00)	0.497
Therapies			
Diet and exercise	1 (14.29)	2 (15.38)	0.730
Oral antidiabetic agents	2 (28.57)	4 (30.77)	0.664
Insulin	3 (42.85)	5 (38.47)	0.608
Oral antidiabetic agents and insulin	1 (14.29)	2 (15.38)	0.730
Chronic complications			
Macrovascular complication	2 (28.57)	5 (38.47)	0.526
Nephropathy	3 (42.85)	1 (7.69)	0.101
Retinopathy	2 (28.57)	0 (0)	0.111
Neuropathy	1 (14.29)	3 (23.08)	0.561

Continuous data with nonnormal distribution are presented as median (first quartile, third quartile), and noncontinuous data are presented as number (%).

**Table 4:** Sleep characteristics of OSA and T2DM patients.

Parameters	Mild-to-moderate OSA (*N* = 7)	Severe OSA (*N* = 13)	*P*
TST (min)	378.71 ± 43.89	398.53 ± 54.83	0.422
Sleep efficiency (%)	78.90 ± 8.36	86.37 ± 9.34	0.094
Stage N1/TST (%)	10.60 ± 4.43	27.13 ± 13.50	0.006^∗^
Stage N2/TST (%)	69.17 (57.23, 72.35)	55.93 (48.00, 71.81)	0.166
Stage N3/TST (%)	2.81 (0, 8.33)	0 (0, 0.05)	0.036^∗^
Stage REM/TST (%)	17.45 ± 4.02	15.10 ± 4.90	0.292
AHI (events/h)	19.13 ± 7.38	62.68 ± 19.06	≤0.001^∗^
N-REM AHI (events/h)	18.27 ± 8.42	63.40 ± 21.26	≤0.001^∗^
REM AHI (events/h)	23.24 ± 16.28	55.42 ± 14.54	≤0.001^∗^
Nadir SpO_2_ (%)	86.00 (85.00, 86.00)	61.00 (42.50, 71.00)	0.001^∗^
Mean SpO_2_ (%)	96.00 ± 1.04	88.96 ± 5.20	0.003^∗^
ODI (events/h)	15.01 ± 6.36	64.80 ± 23.67	≤0.001^∗^

Continuous data with normal distribution are presented as mean ± SD, and continuous data with nonnormal distribution are presented as median (first quartile, third quartile). TST: total sleep time; AHI: apnea-hypopnea index; REM: rapid eye movement; N-REM: nonrapid eye movement; SpO_2_: arterial oxygen saturation; ODI: oxygen desaturation index (^∗^*P* < 0.05).

**Table 5:** Biochemical parameters of OSA with T2DM patients.

	Mild-to-moderate OSA (*N* = 7)	Severe OSA (*N* = 13)	*P*
HbA1c (%)	8.71 ± 1.57	8.41 ± 1.38	0.657
Glucose I (mmol/L)^a^	7.10 (6.86, 8.01)	7.33 (6.47, 7.01)	0.811
Glucose II (mmol/L)	12.00 ± 3.18	11.25 ± 1.82	0.507
Insulin (pmol/L)	61.87 ± 22.36	127.82 ± 57.99	0.010^∗^
C-peptide I (nmol/L)	0.73 ± 0.20	1.00 ± 0.58	0.239
C-peptide II (nmol/L)	1.56 (1.28, 3.79)	1.56 (1.23, 3.92)	0.905
HOMA-IR	2.78 (2.20, 2.93)	4.93 (3.73, 8.37)	0.013^∗^
Cholesterol (mmol/L)	4.60 ± 0.70	4.36 ± 1.21	0.636
Triglycerides (mmol/L)	2.21 ± 0.95	2.35 ± 0.93	0.760
HDL cholesterol (mmol/L)	1.06 ± 0.23	1.07 ± 0.16	0.882
LDL cholesterol (mmol/L)	3.00 ± 0.63	2.76 ± 0.96	0.575
CRP (mg/L)	3.30 (3.28, 4.16)	3.30 (3.29, 3.90)	0.656
TNF-*α* (ng/L)	226.50 (203.00, 327.50)	377.00 (195.00, 3099.25)	0.501
IL-6 (ng/L)	9.00 (6.00, 30.00)	18.50 (12.00, 40.50)	0.165
Cortisol (pmol/L)	272.03 ± 52.24	194.46 ± 51.75	0.005^∗^
Growth hormone (ng/mL)	0.09 (0.07, 0.13)	0.134 (0.09, 0.28)	0.096

Continuous data with normal distribution are presented as mean ± SD, and continuous data with nonnormal distribution are presented as median (first quartile, third quartile). HOMA-IR: homeostasis model assessment of insulin resistance; HDL: high-density lipoprotein; LDL: low-density lipoprotein; CRP: C-reactive protein; TNF-*α*: tumor necrosis factor-*α*; IL-6: interleukin 6 (^∗^*P* < 0.05). ^a^I refers to fasting and II refers to 120 min after 100 g steamed bread meal load.

## Data Availability

The data used to support the findings of this study are included within the article, which are available from the corresponding author upon request.
